# A randomised, double-blind, placebo-controlled parallel trial of closed-loop infraslow brain training in food addiction

**DOI:** 10.1038/s41598-018-30181-7

**Published:** 2018-08-03

**Authors:** Sook Ling Leong, Sven Vanneste, Joyce Lim, Mark Smith, Patrick Manning, Dirk De Ridder

**Affiliations:** 1 Section of Neurosurgery, Department of Surgical Sciences, Dunedin School of Medicine, University of Otago, Dunedin, New Zealand; 2 School of Behavioral and Brain Sciences, University of Texas, Dallas, USA; 3 Neurofeedback Therapy Services of New York, New York, USA; 4 Department of Medicine, Dunedin School of Medicine, University of Otago, Dunedin, New Zealand

## Abstract

The posterior cingulate cortex (PCC) is involved in food craving in obese food addicted individuals. This randomised, double-blind, placebo-controlled parallel study explored the potential therapeutic effects of infraslow neurofeedback (ISF-NF) on food craving targeting the PCC in obese women with symptoms of food addiction. Participants received six sessions of either ISF-NF (n = 11) or placebo (n = 10) over a three-week period. There were no reported adverse effects. Electrophysiologically, there were significant increases in infraslow activity (p = 0.0002) and infraslow/beta nesting (p < 0.001) in the PCC in the ISF-NF group (mean r = 0.004 ± 0.002) compared to placebo (mean r = 0.02 ± 0.002) two days after the last intervention. Also, there was a significant decrease in different dimensions of state food craving compared to baseline and to placebo. Findings suggest that source localized IFS-NF results in electrophysiological changes and may be associated with reduced food craving. This trial is registered at www.anzctr.org.au, identifier, ACTRN12617000601336. This study was funded by the Otago Medical Research Grant: CT375.

## Introduction

Obesity continues to be a significant public health concern with the World Health Organisation estimating in 2016 that globally, 1.25 billion adults were overweight and 650 million were obese^1^. Lately, the term food addiction, defined as the intense persistent desire or ‘craving’ to eat has increasingly been used when discussing the overweight and obesity epidemic^2,3^. Neuroimaging studies have confirmed that the brain’s reward system is accountable for hedonic over-consumption of both food and drugs^3^. The rewarding properties of high fat and sugar foods, activate the mesolimbic dopamine pathways in a similar manner to addictive substances (drugs, alcohol), leading to maladaptive self-control and dysregulation of food intake^4^.

The Yale Food Addiction Scale (YFAS) is a well validated tool used to identify individuals who display signs of food addiction similar to the Diagnostic and Statistical Manual of Mental Disorder V (DSM-V) for substance addiction^5^. Recent research has shown that compared to lean individuals, obese individuals with low (<3) or high (>3) YFAS score share a common pathological increased beta activity in certain regions of the brain including the posterior cingulate cortex (PCC)^6^.

The PCC together with the precuneus, medial prefrontal cortex, medial temporal lobe and inferior parietal cortices make up the default mode network (DMN)^7^. The DMN is considered a self-referential network^8^ where the PCC serves as its hub and is one of the most densely connected brain regions^9^. It has been postulated that the key function of the PCC is to permit the adaption of the self to a changing internal (e.g. allostatic mechanism) and external (e.g. food cues) environment^10^. It could be proposed that among individuals with obesity, the state of being obese is the default neurobiological reference, resulting in higher energy needs^11^. In addition, studies have suggested that communication from the self-referential network to areas involved in intake or suppression of food may be dysfunctional^11,12^.

In reference to addiction, the relationship between PCC activity and craving has been described in many aspects of craving including drug^13^, alcohol^14^, smoking^15^, and food^16^. In a cocaine addiction study, it was reported that the PCC was activated when videos of men smoking cocaine was shown to cocaine users in comparison to non-users^13^. In one case-report, lesioning the PCC destructed the patient’s nicotine addiction^15^. With regards to food craving, thinking of long-term cost of palatable food consumption and craving suppression reduced PCC activity^16^. Taken together, these studies suggest the PCC as a hub in the regulation of different features of craving.

Theoretically, the inflexibility of the DMN towards alterations in energy dynamics could be a reason for the heightened craving for food among some obese individuals, particularly those with symptoms of food addiction^6^. Results from our exploratory study showed that activation of the DMN including the PCC at satiety is present in lean but not obese individuals^6^. Recently, studies have shown that a non-invasive neuromodulation technique, neurofeedback, can induce behavioural changes by targeting specific cortical brain regions^17,18^. During a neurofeedback session, individuals learn to self-regulate and reinforce/reduce brain activity patterns (i.e., operant conditioning) by receiving continuous real-time feedback through the use of EEG recording^10,19^, or fMRI^20^.

There is a growing interest regarding the effectiveness of alpha/theta neurofeedback (i.e., increase theta over alpha power) in the treatment of eating disorders (e.g., anorexia nervosa)^21^, and other substance-related addictive disorders^22^. One study has provided evidence that alpha/theta neurofeedback training of Pz can decrease strong food craving for up to 4 months in non-clinical individuals with overeating^23^. Alpha/theta neurofeedback protocols focus on raising the theta to alpha ratio during relaxation. Given that an increase in theta over alpha power in frontal brain regions is associated with better tolerance to stress, it follows that the alpha/theta protocol enhances an individual’s ability to cope with cravings through increased inhibitory control (i.e., ‘top-down’ approach)^23^. In theory, targeting the PCC would be considered a ‘bottom-up’ approach given that this brain region may be a key hub underlying craving. Here, we use a novel approach; infraslow neurofeedback that may be effective for modulation of PCC activity and craving. Studies have shown that infraslow frequencies typical for the fMRI BOLD signal correlate with infraslow frequencies recorded by EEG^24–26^, and might integrate information from different functional modules^27^. Indeed, it has been reported that the PCC incorporates information from other brain networks predominantly at 0.1 Hz^28^. Therefore, we assume that infraslow neurofeedback, (i.e. operant conditioning of the PCC) may reset allostatic predictive reference resetting, which is considered the drive for overconsumption of food^6,11^, and/or communication via functional connectivity or cross frequency coupling between areas involved in craving related food intake.

Infraslow neurofeedback (ISF-NF) is a recent development in neurofeedback training, focusing on modulating slow wave activity (0–0.1 Hz). To our knowledge this is the first randomised double blind placebo controlled study evaluating the effect of infraslow neurofeedback. In clinical practise, it has been previously reported that training using the infraslow band resulted in a significant reduction of behavioural disruptions, an improved ability to sustain attention during class, and a reduction or elimination of psychotropic medication among children with Emotional Disorder and Pervasive Developmental Disorder^29^.

The main aims of this study are to verify whether infraslow closed loop brain training 1) is technically feasible, and 2) results in measurable changes in brain activity. In addition, we investigated whether ISF-NF has an effect on food craving scores.

## Methods

### Participants

Participants between the ages of 18 to 60 years were recruited from advertisements in local newspapers and on notice boards with an invitation to participate in a potential therapeutic method to curb food craving. Interested individuals were invited to the BRAI3N neuromodulation clinic of the University Hospital of Otago, Dunedin, New Zealand for a screening procedure. All eligible participants had to score 3 or more on the YFAS^5^ and had to have a body mass index (BMI) that is equals to or above 25. Exclusion criteria included: (1) major weight gain or loss (>5 kgs) in the last 6 months; (2) recent significant head injuries; (3) females who are or intend to become pregnant; (4) co-morbidities associated with obesity (e.g., diabetes, obstructive sleep apnoea); (5) centrally active medications; or (5) neurological or psychiatric disorders.

Given the small sample size of a pilot study design, we included only females, because dysfunctional eating behaviours are reported to be more prevalent among women compared to men in the general population^30^. All participants gave written informed consent to participate and were paid NZD 100 at completion of the protocol. This study was performed in accordance to the Helsinki declaration standards and was approved by the Northern B Health and Disability Ethics Committee (17/NTB/61). The protocol was prospectively registered at www.anzctr.org.au on the 27^th^ of April 2017, identifier, ACTRN12617000601336.

Our previous pilot study^31^ investigating the effect of transcranial pink noise stimulation on food craving in obese women showed that 50% of obese participants meet the criteria. Thus, it was estimated that around 40 women will need to be screened.

To ensure that sample size is balanced across the two groups over time, block randomisation was applied. Given the small sample size, a block size of four was used. A researcher from the group who has no direct contact with the participants conducted the randomisation process using the program on randomization.com. This tool is a valid randomisation program utilised by clinical trial researchers.

**Figure 1:**
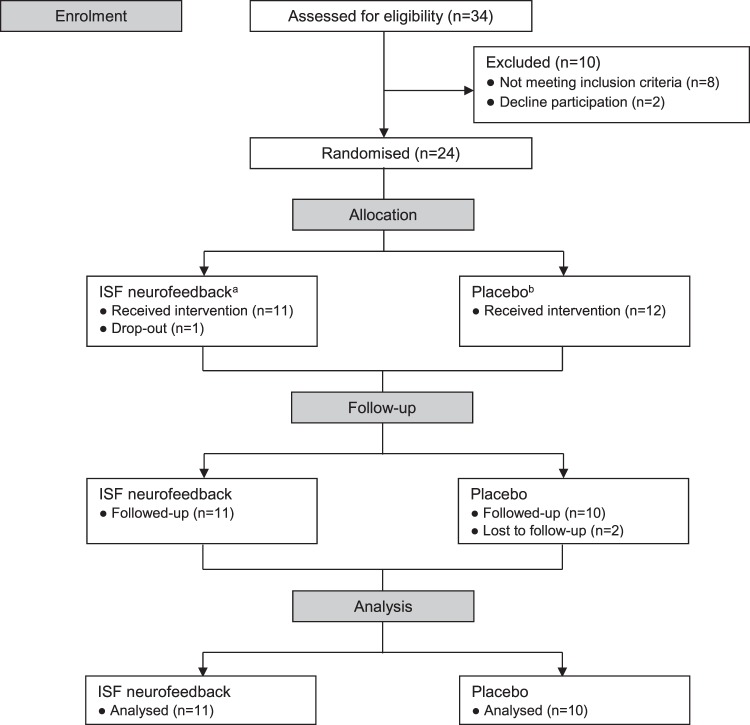
Study flow-chart showing participant selection, randomisation to two study groups, and number of participants completing the study. (**a**) Six sessions of infraslow neurofeedback (ISF-NF) targeting the posterior cingulate cortex (PCC). (**b**) Six sessions of placebo (simulation) ISF neurofeedback.

Thirty four women responded to the advertisement and were assessed for eligibility. Eight women did not meet the inclusion criteria and two declined to participate. Twenty four women were randomised to either ISF-NF (n = 12) or placebo (n = 12). One participant dropped out in the ISF-NF group before the end of treatment and two participants in the placebo group were lost to follow-up. A participant flow-chart according to CONSORT-guidelines is presented in Fig. 1 and sample characteristics are described in Table 1. Of the 21 women included in the final analysis of the study, one woman in each group identified as Māori while all other participants identified as New Zealand European.

**Table 1:** Comparison of baseline demographic and behavioral variables between the ISF-NF group and placebo group.

Variables	ISF –NF n = 11 *mean (SD)*	Placebo n = 10 *mean (SD)*	P value^a^
Age (years)	44.0 (13.2)	42.0 (14.7)	0.752
Height (cm)	166.5 (5.8)	164.6 (6.8)	0.511
Weight (kg)	92.3 (19.1)	90.8 (19.4)	0.864
BMI^b^ (kg/m^2^)	33.6 (8.5)	33.4 (6.2)	0.845
YFAS^c^	4.9 (1.6)	5.1 (1.4)	0.772
**FCQ-S** ^**d**^			
*Intense desire to eat*	3.8 (1.0)	3.8 (0.6)	0.967
*Anticipation of relief from negative states*	3.6 (0.7)	3.4 (0.9)	0.744
*Craving as a physiological state*	3.2 (0.9)	3.2 (0.7)	0.830
*Anticipation of positive reinforcement*	3.5 (0.8)	3.5 (0.8)	0.959
*Lack of control over eating*	3.6 (1.2)	3.6 (0.8)	0.995

^a^Independent t-test between two groups.

^b^Body Mass Index.

^c^Yale Food Addiction Scale. Calculated on a continuous scale, where a score of more than 3 indicates symptoms of high food craving.

^d^Food Craving Questionnaire- State. Consists of 5 subscales, assessing different dimensions of situational food craving.

### Study design and procedures

The study was a four-week, randomised, double-blind, parallel trial. Different researchers conducted the EEG assessments/craving status to those carrying out the treatments. All researchers who had contact with participants were blinded to treatment allocation to minimise possible bias. All patients were blinded to treatment assignment.

Participants were randomised to either ISF-N training or placebo. At pre-treatment (T0), food craving was measured using the validated Food Craving Questionnaire (FCQ-S)^32^ and resting state brain activity sampled using EEG. Height was measured without shoes to the nearest 0.5 cm using a stadiometer and body weight assessed using a Bioelectric Impedance Analysis (BIA) machine (BC-418, Tanita Co., Tokyo, Japan).

Participants received either ISF-NF or placebo three times a week for two weeks totalling up to six sessions. The first training session was for 10 minutes and the subsequent 5 sessions were 20 minutes each. Two-days after the last treatment session (T1), participants were asked to complete the same battery of questionnaires and to perform another resting state EEG. Follow-up assessments were only conducted at one time point as there is a high participant burden associated with this trial.

### ISF-NF neurofeedback and placebo

ISF-NF and placebo ISF-NF were administered with participants sitting in a comfortable chair with their eyes closed. After careful skin preparation, the appropriate Comby EEG (Ag/AgCl) cap was placed on the participant’s head with reference electrodes at the mastoids. The impedances of the active electrodes were kept below 5 kΩ. Before the training period, participants were instructed to relax and listen to the sound being played. For the ISF-NF group, a distinct tone was used for ISF reinforcement at the PCC. Reward threshold was adjusted in real time at above 90%. In other words, for 90% of the time, a sound was played (reward) when participant’s brain activity meets the infraslow magnitude (threshold). For the placebo ISF-NF group, the simulation protocol by Brainmaster Inc. was administered. The simulation protocol played a sound at random.

Before the first training session, a simple explanation was given to participants. They were informed that research has shown that the brain of individuals with a BMI of more than 25 functions a little differently from normal-weight individuals and that we were trying to train their brains to normalize. The sound they hear during neurofeedback reflects whether they were doing well.

### Assessments

#### Screening: Yale Food Addiction Scale (YFAS)

The YFAS^5^ is a well validated 27 item questionnaire used to identify those who display signs of food dependency similar to the DSM-IV characteristics of substance addiction. The YFAS was computed as a continuous scale from 0–7 with participants scoring three or more included in this study as showing symptoms of food addiction.

#### Primary outcome: EEG and source localisation

Resting state EEG was sampled continuously at 500 Hz using a Mitsar-EEG 202 DC amplifier. Briefly, EEG was recorded with 19 electrodes (Fp1, Fp2, F7, F3, Fz, F4, F8, T7, C3, Cz, C4, T8, P7, P3, Pz, P4, P8, O1 O2) according to the 10–20 placement. Linked ears reference were used and all electrode impedances were kept below 5 kΩ. EEGs were recorded with participants sitting upright in a comfortable chair with their eyes closed. All EEGs were recorded for 6 minutes.

Raw data was resampled at 128 Hz, filtered from 0.001 Hz to 44 Hz, plotted, and carefully inspected for manual artefact rejection on EEGLAB. Subsequently, average cross-spectral matrices were computed for bands infraslow (0.01–0.1 Hz), delta (2–3.5 Hz), theta (4–7.5 Hz), alpha (8–12 Hz), beta (12.5–30 Hz) and gamma (30.5–44 Hz).

Standardized low-resolution brain electromagnetic tomography (sLORETA) was used to calculate the intracerebral electrical sources that generated the scalp-recorded activity in each of the six frequency bands. Technical details of sLORETA and its validity have been previously published^33,34^. The sLORETA output (computed as log-transformed current density, A/m^2^) was used in ensuing analyses described below (i.e., whole brain, region of interest, phase to power nesting).

#### Secondary outcome: Food Craving Questionnaire State (FCQ-S)

The FCQ-S^32^ assesses food cravings occurring ‘right now’. The FCQ-S has a total score ranging from 15–75 and consists of 5 subscales: ‘Intense desire to eat’; ‘Anticipation of relief from negative states from food’; ‘Craving as a physiological state’; ‘Anticipation of positive reinforcement from food’; and ‘Lack of control over eating’. The subscale assessed different dimensions of situational food craving.

### Statistical analyses

At T0, independent t-test was used to examine differences between the ISF-NF group and placebo for demographic variables and behavioural assessments (Table 1). Further analyses are explained in detailed below. All statistical analyses were performed using STATA 14 (StataCorp. 2017).

#### Electrical neuroimaging

Analyses were conducted comparing: (1) NF-ISF versus placebo at T0; (2) NF-ISF versus placebo at T1; (3) NF-ISF at T0 versus T1; and (4) placebo at T0 versus T1. Comparisons were not analysed after three session of treatment as only four participants attended those EEG sessions.

Whole brain: This sLORETA analysis is a non-parametric, null hypothesis comparison and is based on estimating the empirical probability distribution for max-statistic through randomisation. It corrects for multiple testing, and due to its non-parametric nature, does not rely on any assumption of Gaussianity^35^. The significant thresholds of sLORETA’s statistical contrast maps were calculated through multiple voxel-by-voxel comparisons and was based on 5000 permutations.

ROI: In this analysis, log-transformed current density was averaged across all voxels belonging to the PCC for the 6 bands of interest. We applied a repeated measures MANOVA comparing the two groups, pre- and post- treatment. If there was a significant main effect, a univariate ANOVA was conducted to compare differences for each frequency band.

Phase to Power Nesting: It has been suggested that beta activity governs the ‘status quo’ of the DMN at resting state^11^. Given that the PCC communicates with other brain regions at infraslow frequencies^28^, it follows that infraslow-beta coupling would be an effective way of communication between the PCC and cortically distant brain regions. In this analysis, we examined whether there was evidence of increased infraslow beta coupling in the PCC in the ISF-NF group compared to placebo after treatment. Technical details on phase to power analysis have been previously described by the researchers^36^. A repeated measures ANOVA was performed on the final output (Pearson’s correlation between the infraslow component and the envelope of beta) to assess the differences in pre- and post-treatment in phase to power coupling for the two groups.

#### Food Craving Questionnaire State (FCQ-S) and correlation analysis

Repeated measures ANOVA was conducted with groups as between-subject variable, time as a repeated factor (T0, T1), and the FCQ-S scores as dependent variable. Further analysis using pairwise comparison was carried out to test significant differences between the different time points. In addition, pairwise correlation analysis was conducted between log-transformed infraslow current density in the PCC with FCQ-S sub-scales that had a main effect.

### Data availability

The datasets generated during and/or analysed during the current study are available from the corresponding author on reasonable request.

## Results

### Whole brain

**Figure 2:**
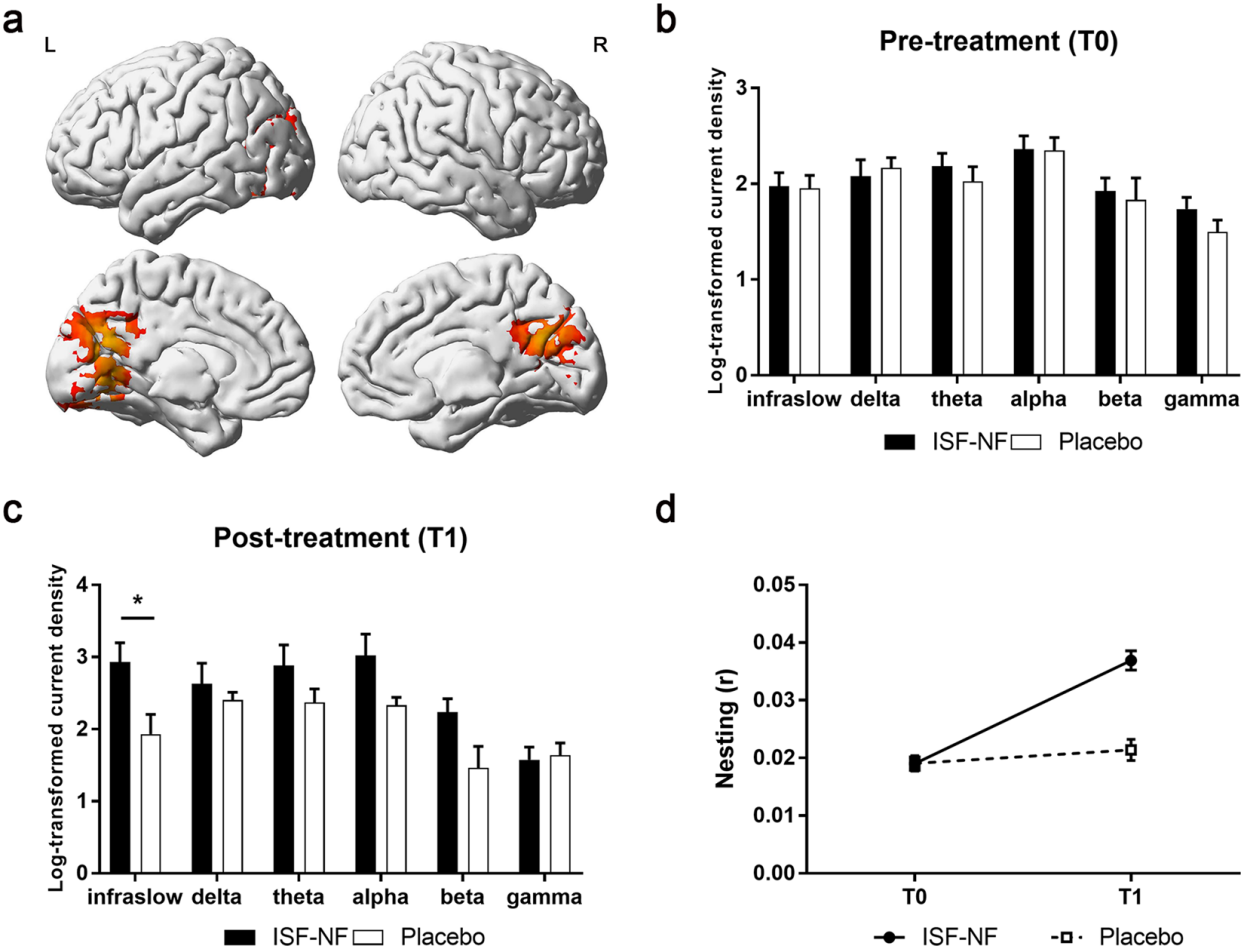
(**a**) Significant increase in infraslow activity in the posterior cingulate cortex (PCC) in the ISF-NF group compared to placebo after treatment (T1) (p = 0.040) as well as in the ISF-NF group at T1 compared pre-treatment (T0) (p = 0.0002). Red colour represents an increase in activity. (**b**,**c**) A region of interest analyses using univariate modelling of the posterior cingulate cortex (PCC) pre- (T0) and post-treatment (T1) showed a significant difference for the infraslow band, F(1, 19) = 5.25, p = 0.034 in the ISF-NF group compared to placebo at T1. (**d**) An independent t-test comparing infraslow/beta nesting (r) in the posterior cingulate cortex (PCC) post-treatment (T1) shows a significant increase in infraslow/beta nesting for the ISF-NF group (mean r = 0.04 ± 0.002) compared to.

After correcting for multiple comparisons, there were no significant changes in resting state whole brain analysis when comparing 1) NF-ISF versus placebo at T0; and 2) placebo at T0 versus T1. At T1, there was a significant increase in infraslow activity in the PCC, in the ISF-NF group compared to placebo (Fig. 2a). Also, there was an increase in the PCC for the beta band in the ISF-NF group compared to placebo (p = 0.05) at T1. No significant effect was obtained for the delta, theta, alpha and gamma bands at T1 when comparing the ISF-NF group and placebo.

When compared to T0, there was a significant increase in infraslow activity at T1 in the ISF-NF group (Fig. 2a). No significant effect was observed for the delta, theta, alpha, beta and gamma bands.

### ROI

Repeated measures MANOVA showed there was a significant main difference between ISF-NF and placebo on the different frequency bands over time, F(6, 14) = 3.04, p = 0.041; Wilks’ Λ = 0.434, partial η2 = 0.462. Univariate tests indicated the intervention effect was for the infraslow band, F (1, 19) = 5.25, p = 0.034. For this band, there was an increased in log-transformed current density in the PCC for the ISF-NF compared to placebo (Fig. 2b,c).

### Phase to power nesting

Repeated measures ANOVA showed that the treatment-by-time interaction was significant F(1,19) = 32.67, p < 0.001 (Fig. 2d). To verify the source of this interaction, further analyses were conducted. At T1, independent t-test showed a significant increase in infraslow/beta nesting for the ISF-NF group (mean r = 0.04 ± 0.002) compared to placebo (mean r = 0.02 ± 0.002), p < 0.001. Also, for the ISF-NF group, paired t-test showed a significant increase in infraslow/beta nesting at T1 (mean r = 0.004 ± 0.006) compared to T0 (mean r = 0.02 ± 0.004), p < 0.001.

### FCQ-S and correlation analysis

**Figure 3:**
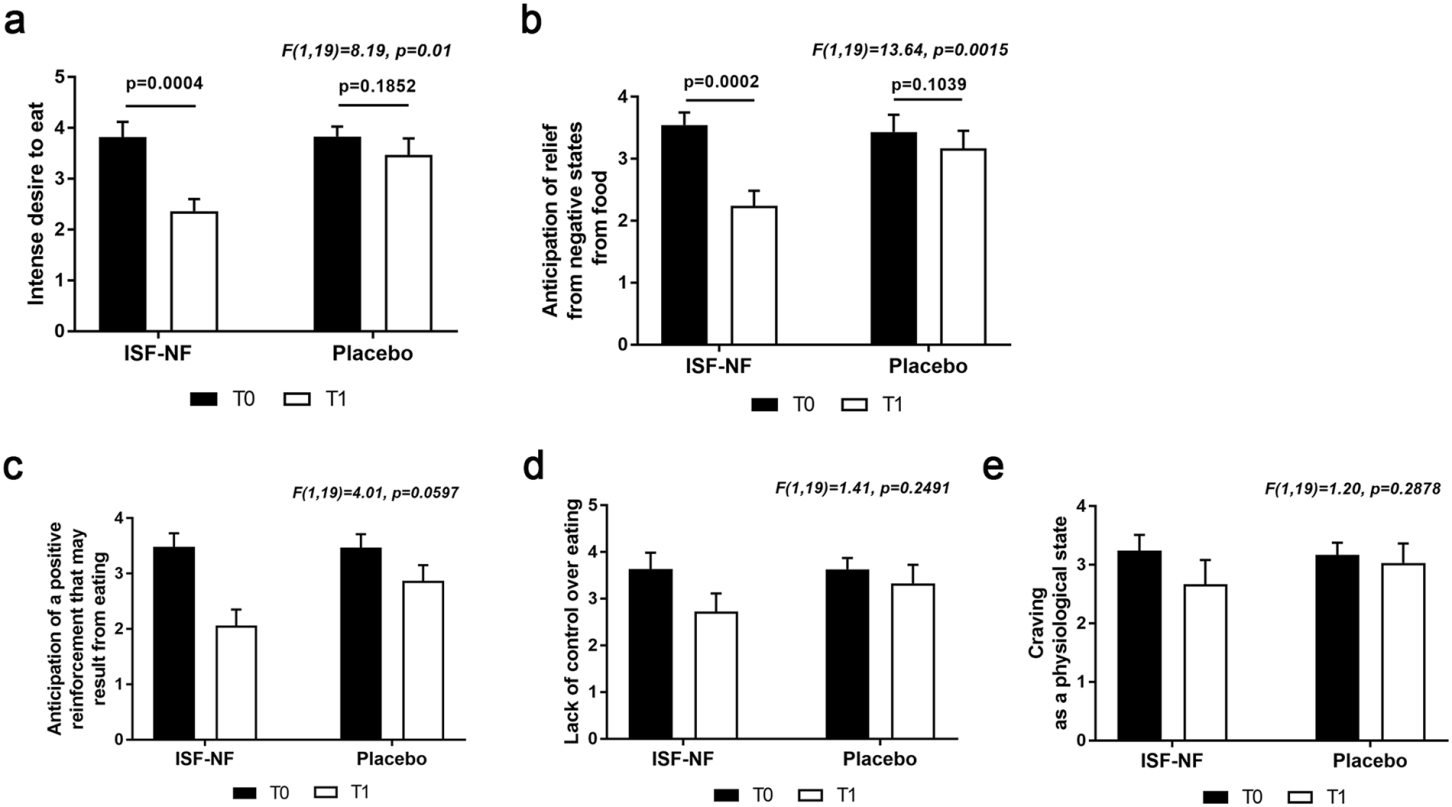
A repeated measures ANOVA reveals significant main effects for two of the five FCQ-S subscales, a) intense desire to eat, F(1,19) = 23, p = 0.01 and b) F(1,19) = 13.6, p = 0.0015. Paired t-test shows that there is a significant decrease in the ISF-NF group post-treatment (T1) compared to pre-treatment (T0) for the intense desire to eat subscale, p = 0.0004 (**a**) and the anticipation of relief from negative states from food subscale, p = 0.0002 (**b**).

A repeated measures ANOVA revealed significant main effects for two of the five FCQ-S subscales, indicating that craving ‘right now’ was decreased after treatment in comparison to before treatment. The results for the significant FCQ-S subscales are presented in Fig. 3.

Further exploration of the data using paired t-test showed that there was a 39% decrease in intense desire to eat **(**Fig. 3a), and a 36% decrease in anticipation of relief from negative states from eating (Fig. 3b) in the ISF-NF group at T1 compared to T0.

There were no significant correlations between log-transformed current density of the infraslow band in the PCC with the ‘intense desire to eat’ and the ‘anticipation of relief from negative states from eating’ sub-scales.

### Adverse effects and integrity of blinding

To our knowledge, this is the first clinical trial that recorded possible adverse effects of ISF-NF. Guided by previous published alpha/theta neurofeedback studies, at the beginning of each session, participants were asked if they experienced any: (1) headaches; (2) tiredness; (3) nightmares; or (4) confusion. Participants were also encouraged to report any side effects that they felt may be a result of treatment.

In the NF-ISF group, 5 participants reported having ‘weird dreams’ during the 4^th^ session and another 2 participants during the 5^th^ session. When asked to elaborate, the women described their dreams as vivid or unusual. They also stated that they clearly recall their dreams upon waking up in the morning. However, it was reported that they felt more rested than usual despite having uncanny dreams during sleep.

At T1, participants were asked ‘Which condition do you think you received?’ Two women in the NF-ISF group failed to correctly identity their allocation group. As for the 9 participants in the ISF-NF group who guessed correctly, the two main reasons for their answer were improvement in craving symptoms and feeling more rested the following day. All participants in the placebo group correctly identified their allocation group.

## Discussion

This is the first randomised double blind placebo controlled study evaluating the effect of ISF-NF. The main aims of the study were to investigate whether ISF-NF was feasible and can result in measurable brain activity changes. Electrophysiological results showed significant increases in infraslow activity and infraslow/beta nesting in the PCC in the ISF-NF group compared to placebo two days after the last intervention. Moreover, our preliminary findings indicate that an increase in infraslow activity in the PCC among individuals with symptoms of food addiction may translate to a significant reduction in different dimensions of food craving as measured by the FCQ-S.

Interestingly, there were no significant correlations between changes in food craving measures and infraslow brain activity in the PCC. These results suggest the possibility of an indirect effect of ISF-NF on food craving. Given that the PCC is the integrating hub of self-referential thoughts and activities^8^, an increased interoceptive awareness after ISF-NF may have resulted in better identification of internal appetite signals. Studies have shown that poor interoceptive awareness is a key feature of eating disorders^37^, and this interpretation of study results warrant further examination in future ISF-NF studies.

Importantly, the current results expand on previous studies by identifying a possible target area to re-train brain circuits associated with food addiction, and potentially other addictive disorders, as craving in addiction is generated by a common circuit, involving the anterior, mid and posterior cingulate as well as the nucleus accumbens^38,39^. It has also been proposed that connectivity between the craving areas predicts the time to relapse, and the stronger the PCC connectivity, the longer the time to relapse^40^. Theoretically, each of these areas of the craving circuit could be used as a target for neuromodulation. For example, results from our previous stimulation (transcranial pink noise stimulation) study showed that reduction in food cravings can also be achieved by targeting the rostral anterior cingulate cortex^31^.

One possible advantage of this ISF-NF protocol compared to the theta/alpha protocol is that it targets the underlying drive to overeat using a ‘bottom-up’ strategy, focusing on reducing the salience attached to food and therefore the urge to overeat. This may allow successful control of food provoked by environmental cues. Previous neurofeedback studies in food and alcohol addictions^21–23^ using the alpha/theta protocol focused on increasing inhibitory control via the ‘top-down’ approach. Among overweight and obese individuals, ‘top-down’ strategies are often used to try and control, resist or distract from behaviours that lead to overeating^41^. These types of nutritional or behavioural strategies resulting in a caloric deficit are often not successful in the long term as with dramatic weight loss, hormonal and neural changes increase the desire to eat^42^. This often results in the individual not only regaining the lost weight, but gaining weight beyond that of the original start point^42^.

It is possible that infraslow activity may be a ‘carrier wave’ for higher frequency waves, such as beta via nesting or cross-frequency coupling. Also, there is growing evidence that the PCC may integrate information from other brain regions at infraslow frequencies^28^ and that beta activity maintains the existing state or ‘status quo’ of the DMN^7,8^. In both food-addicted and non-addicted obesity, the PCC differs from lean controls by the presence of increased gamma activity^6^, which represents a ‘bottom-up’ prediction error, whereas beta usually represents a ‘top-down’ prediction^41,43^. Hypothetically, this infraslow-beta coupling could represent a regained ‘top-down’ control by maintaining a status quo of food intake. This communication model is supported by results of the current study, as there was an increase in infraslow/beta nesting after ISF-NF but not after placebo treatment. Also, our results suggest that operant conditioning with neurofeedback may ‘decouple’ and normalize infraslow/beta nesting.

It is of note that this study was not designed to investigate the long term effects of ISF-NF, but to verify whether ISF-NF is feasible. Although studies have reported that trait and state food cravings are interdependent^44^, a larger ISF-NF intervention trial with multiple follow-ups has to be conducted to warrant confirmation on whether current experiences in decreased physiological state cravings represents a habitual stable trait.

Taking into account the nature of a feasibility study, several important limitations are noteworthy. Firstly, there was a lack of weight assessment post-intervention. Given that this is the first ISF-NF study, there was a concern of the possibility that being aware that weight is a study outcome would affect subjective reporting of food craving and eating behaviour. The question of whether the observed reductions in food craving using this ISF-NF parameter can translate to weight change will need to be examined in a weight-loss intervention with a longer follow-up. Secondly, meal time and diet were not taken into account during neurofeedback sessions which theoretically, may have influenced participants’ motivation to self-regulate brain activity. Nevertheless, all ISF-NF sessions and assessments for each participant were conducted at the same time of the day, suggesting that any within-subject changes would have been consistent over time. In addition, at a group level, there were no significant effects in the placebo group compared to the ISF-NF group suggesting that changes were treatment related. In future studies using this protocol, meal time and diet should be addressed to understand their impact on ISF-NF. Thirdly, we did not assess menstrual cycle phases during pre- and post- follow-up assessments. Studies have shown that women exhibit greater cravings for high fat and sugar foods during menstrual flow compared to other stages of the cycle^45^. However, given that this is a four –week intervention, pre- and post- food craving assessments for each woman would have been conducted in the same menstrual cycle phase. Lastly, the small sample size of only female participants limits the generalizability of the results and the applicability and effectiveness of ISF-NF should be examined in a larger female and male sample.

With regards to the integrity of the blinding procedure, we found that participants were accurate in guessing their allocation group, and the main reason related to correct prediction was clinical response (i.e., decrease in cravings and feelings of being more rested). Given that the placebo group was also accurate in their prediction, it could be established that women attributed their physical and mental improvements to ISF-NF, instead of treatment expectancy (i.e., women who believed that they receiving ISF-NF expected therapeutic outcomes). Moreover, none of the participants in the study had prior experience with any neurofeedback protocols.

In conclusion, these preliminary findings show that ISF-NF targeting the PCC can alter brain activity and suppress food craving in individuals with overweight or obesity who show signs of food addiction. It would be a worthwhile exercise to conduct further research to replicate these positive findings. Given the increasing prevalence of obesity and the low success rate of weight loss interventions, there is an urgent need to develop new approaches that may complement behavioural strategies for weight management.
